# An Oxidized Squalene Derivative from *Protium subserratum* Engl. (Engl.) Growing in Peru

**DOI:** 10.3390/molecules17067451

**Published:** 2012-06-15

**Authors:** John Lokvam, Paul V. A. Fine

**Affiliations:** 1 Department of Biology, University of Utah, 257 S 1400 E, Salt Lake City, UT 84112, USA; 2 Department of Integrative Biology, University of California, 1005 Valley Life Sciences Building 3140, Berkeley, CA 94720, USA

**Keywords:** *Protium*, defense chemistry, habitat specialization, squalene

## Abstract

*Protium subserratum* (Burseraceae) is a neotropical tree species that is comprised of several habitat-specific ecotypes having distinct defense chemical profiles. A previously unknown triterpene, 25,30-dicarboxy-26,27,28,29-tetraacetoxy-10,11,14,15-tetrahydrosqualene, was isolated from *P. subserratum* young leaf tissue of one ecotype growing in Peru. The structure of **1** was determined by spectroscopic study, including 1 and 2D nuclear magnetic resonance experiments.

## 1. Introduction

**Figure 1:**
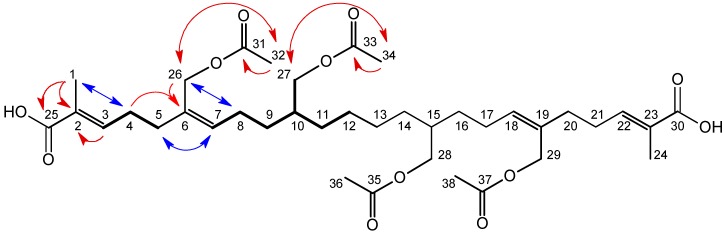
25,30-dicarboxy-26,27,28,29-tetraacetoxy-10,11,14,15-tetrahydrosqualene (**1**) from *Protium subserratum* young leaf tissue. Scalar coupling networks (bold bonds) and pertinent HMBC and NOESY correlations (red and blue arrows respectively) are indicated for one mirror half of the molecule.

*Protium subserratum* Engl. (Engl.) (Burseraceae) is a Neotropical tree species belonging to the frankincense and myrrh family. It is widely distributed in lowland rainforests in South America, where it grows on a variety of soil types, ranging from nutrient-poor white sand to relatively nutrient-rich clay [1]. Because of this, *P. subserratum* is considered a habitat generalist. Nevertheless, ecotypes of this species that have distinct morphologies and phylogenetic histories [2] are associated with the different soil types and can be found growing just meters apart. It is not known what factors promote and maintain habitat specialization in this taxon. A recent field experiment, however, showed that insect herbivore communities interact with soil types to reinforce habitat boundaries [3]. As part of a larger investigation of plant-herbivore interactions and the diversification of plant lineages, we are examining leaf secondary metabolite chemistry in white-sand and clay-soil ecotypes of *P. subserratum* that grow in Amazonian Peru. We focus on young, expanding leaves because they are the preferred food source of most herbivorous insects [4] and because their defense chemical profiles are often markedly different than mature leaves of the same plant [5,6]. In this paper, we report on the structure of a previously unknown squalene derivative (Figure 1) that accumulates primarily in the leaves of the *P. subserratum* ecotype growing on white-sand soils.

## 2. Results and Discussion

Compound **1** (Figure 1) was isolated from dried, ground *P. subserratum* young-leaf tissue following flash chromatography fractionation of the 80% ethanol leaf extract and HPLC purification. The high resolution FTICR mass spectrum of **1** gave an [M + H]^+^ ion of *m/z* 707.40728, indicating a molecular formula of C_38_H_58_O_12_. A proton-decoupled ^13^C-NMR spectrum acquired at 26 °C, however, showed only 16 resonances. When the acquisition temperature was increased, first to 75 and then to 100 °C, 19 resonances were resolved (Table 1). A qDEPT experiment showed five unprotonated carbons, three with shifts indicative of carboxy carbonyl carbons (*δ* 167.7, 169.0 and 169.2) and two with olefinic shifts (*δ* 127.0 and 130.0). DEPT also showed the presence of three methyl, eight methylene and three methine peaks. Six of the methylene carbons had aliphatic shifts while two were oxygen bound (*δ* 60.4 and 65.5). In addition, two of the methine resonances arose from olefinic carbons (*δ* 132.3 and 139.4) and one from an aliphatic carbon (*δ* 35.8). Examination of the ^1^H-^1^H COSY spectrum, with supporting evidence from an HSQC experiment, gave two scalar coupling networks involving three and seven carbons respectively (Figure 1). An HMBC experiment provided the long-range ^1^H-^13^C correlations that permitted assembly of a structure uniquely consistent with the NMR spectroscopic data. This structure, however, possessed a *sp*^3^-hybridized terminal methylene carbon (Figure 1, carbon 12). Moreover, despite the fact that every resonance was accounted for, the compound had exactly half the atoms predicted by the high-resolution mass spectrum. The sum of these data led to the conclusion that **1** was a symmetrical molecule consisting of halves that produced identical NMR spectra, each with a nominal mass of 353 amu.

**Table 1:** ^13^C- and ^1^H-NMR Chemical Shifts ^a^ and ^1^H Multiplicities ^b^ for **1** in DMSO-d_6_, 100 °C.

C number ^c^	Assignment	^13^C (ppm)	^1^H (ppm)	Multiplicity (*J*, Hz)
1, 24	CH_3_	11.1	1.75	br s
2, 23	C	127.0		
3, 22	CH	139.4	6.63	dd (7.2, 7.2)
4, 21	CH_2_	26.1	2,28	m
5, 20	CH_2_	32.6	3.17	dd (7.2, 7.2)
6, 19	C	130.0		
7, 18	CH	132.3	5.44	dd (7.3, 7.3)
8, 17	CH_2_	23.7	2.11	m
9, 16	CH_2_	30.3	1.36	m
10, 15	CH	35.8	1.65	m
11, 14	CH_2_	29.8	1.30	o
12, 13	CH_2_	25.5	1.30	0
25, 30	COOH	167.7		
26, 29	CH_2_	60.4	4.59	br s
27, 28	CH_2_	65.5	3.95	m
31, 37	COOH	169.2		
32, 38	CH_3_	19.5	2.00	s
33, 35	COOH	169.0		
34, 36	CH_3_	19.5	2.01	s

^a^ referenced to the center peak of the solvent multiplet, 39.5 and 2.51 ppm, respectively, for ^13^C and ^1^H; ^b^ br s = broad singlet; dd = double doublet; m = multiplet; s = singlet; o = overlap; ^c^ see Figure 1.

The stereochemistry of the double bonds (Figure 1) in **1** was determined using a 2D-NOESY experiment. In order to reduce the risk of sample degradation, the NOESY experiment, as well as a second ^1^H-^1^H COSY experiment, was acquired at 26 °C. Data from the NOESY experiment were analyzed in the context of proton chemical shifts that were slightly altered as a result of the change in experimental temperature. NOE correlations between the carbon 1 and 4 protons, as well as the absence of any correlation between carbon 1 and 3 protons, established the *E*-configuration for the carbon 2-3 double bond. The presence of NOE correlations between both the carbon 5 and 7 protons and the carbon 8 and 26 protons, as well as the absence of any correlation between the carbon 7 and 26 protons, indicated the *Z*-configuration for the carbon 6-7 double bond. The relative stereochemistry at carbon 10 was not determined and there is no record of this squalene derivative ever having been synthesized. Comparisons to standards were therefore not possible.

**Figure 2:**
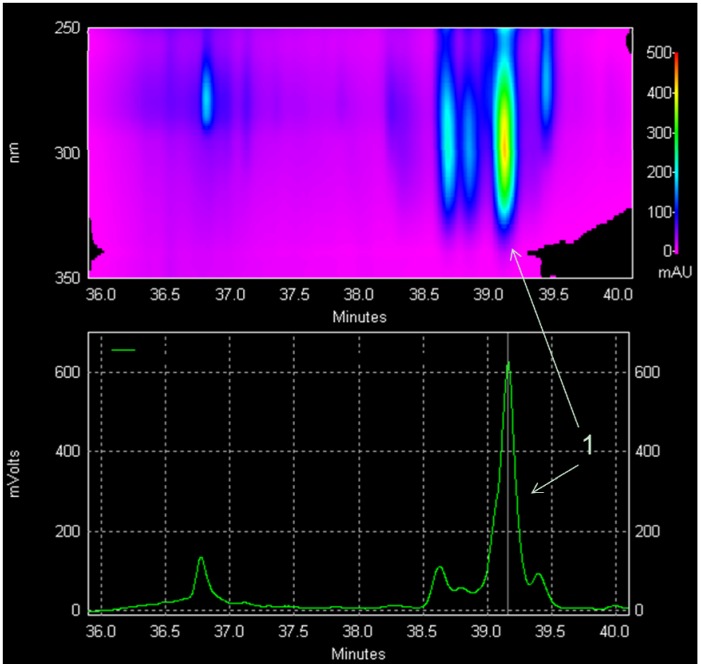
Diode array detector (upper panel) and evaporative light-scattering detector (lower panel) chromatograms from HPLC analysis of the fraction containing **1**. The extract is from a white-sand ecotype of *P. subserratum* growing near Jenaro Herrera, Peru. Based on retention times and UV absorbance, several forms of oxidized terpenes are likely present in the leaves, with **1** being the most abundant. Related forms were not isolated in sufficient quantity to permit structure solution.

Based on chromatographic and UV absorbance properties (Figure 2), *P. subserratum* populations of eastern Peru accumulate several related forms of oxidized terpenes, with **1** being the most abundant. At present, the significance of these metabolites is unknown. But given the fact that **1** comprises at least 0.1% of leaf dry weight, its relatively high concentration points to an allelochemical function. Squalene itself has been shown to be an effective synomone: It is synthesized in apple leaf tissue in response to leaf miner attack and attracts the parasitic wasp *Pholester bicolor* to probe leaves, even when applied to the leaf surface in the absence of any host herbivore [7]. Whether the modifications to terpenoid metabolites that we observe in *P. subserratum* are related to their signalling properties remains to be investigated. For the purposes of the chemical analyses carried out in this study, only undamaged leaves were collected. Thus, the leaf chemistry we characterized more likely represents a constitutive metabolic profile than one generated in response to wounding.

## 3. Experimental

### 3.1. General

Optical rotation was measured on a Perkin-Elmer 343 polarimeter. The UV spectrum was recorded using a Hitachi U-4100 spectrophotometer (Hitachi High Technologies Corp., San Jose, CA, USA). High pressure liquid chromatography (HPLC) analyses were done using a Hitachi LaChrom Elite system. Detection was by photo-diode array (Hitachi L-2450) and/or evaporative light-scattering (Sedex 55, SEDERE, Alfortville, France). Compound purifications were carried out using an Atlantis T3 3μ ODS analytical (4.6 × 150 mm) column (Waters Corp., Milford, MA, USA). Nuclear magnetic resonance (NMR) spectroscopic data were acquired on a Varian iNOVA 500 MHz spectrometer (Agilent Technologies, Santa Clara, CA, USA) at 100 °C, unless otherwise noted. Direct detection probes were used for ^13^C observation. Z-gradient indirect detection probes were used for ^1^H, COSY (^1^H-^1^H correlations), HSQC (1-bond ^1^H-^13^C correlations), HMBC (multiple-bond ^1^H-^13^C correlations) and 2-dimensional NOESY (nuclear Overhauser effect) experiments. Chemical shifts were referenced to the center peak of the DMSO-*d*_6_ solvent multiplet: 39.5 and 2.51 ppm for ^13^C and ^1^H, respectively. Data from high-resolution electro-spray ionization mass spectrometry were obtained using a ThermoElectron LTQ-FT [Fourier transform-ion cyclotron resonance (HRFTICR) measurement; ThermoFisher Corp., Waltham, MA, USA], operated in positive ion mode.

### 3.2. Plant Material

*Protium subserratum* leaves were collected in 2011 at two field sites (white-sand and clay soil forest) near Iquitos, Peru and two field sites (white-sand and clay soil forest) in Jenaro Herrera, Peru. Iquitos and Jenaro Herrera are 100 km distant from one another and are on opposite sides of the Amazon/Ucayali river, which is approximately one km wide in this area. From each of the four populations, we sampled five to nine individuals for leaf chemistry. Plants were identified by P. V. A. Fine. Adult individuals from the same forests have been included in a larger phylogeographic study on this tree species [2]. Voucher specimens are maintained at UC (University Herbarium, University of California Berkeley). Iquitos white-sand collections of *P. subserratum* have numbers IM 1013, 1014 and 1015; Jenaro Herrera white-sand collections have numbers ND 5960, 5961 and 5963 [2]. Leaves were dried in silica gel at room temperature and then returned to the University of Utah for analysis. From each of the four populations, we sampled five to nine individuals for leaf chemistry. Leaves from white-sand populations of *P. subserratum*, (those containing relatively high concentrations of **1** were pooled and submitted to the following extraction and isolation protocol.

### 3.3. Leaf Extraction and Isolation of 25,30-Dicarboxy-26,27,28,29-tetraacetoxy-10,11,14,15-tetra-hydrosqualene *(**1**)*

Once received in Utah, leaves were dried under high vacuum (10^−2^ Torr) for 24 h, then combined and pulverized using a Retsch MM 200 mill (Retsch GmbH, Haan, Germany). Pulverized leaf tissue (9.2 g) was extracted three times with 80% EtOH. The alcohol was removed at low pressure and the remaining aqueous portion was defatted with CHCl_3_, then applied directly to a 2 × 10 cm ODS column that had been equilibrated with 100% water. The column was serially eluted with 100% water (200 mL) and then 60%, 80% and 100% MeOH (200 mL each),. Solvent was removed from each fraction first under low pressure and then under high vacuum for 24 h. A total of 55 mg was obtained from the 80% MeOH fraction. HPLC analysis showed that this fraction contained a relatively high proportion of **1**. This fraction was redissolved in MeOH to give a final concentration of 100 mg/mL. An HPLC separation of **1** was optimized using CH_3_CN (A) and water containing 0.1% formic acid (B) as the elution solvents. An isocratic mixture of 60% A in B was programmed for 14 min followed by a 5 min wash (100% A) and column reequilibration. The flow rate was 1 mL/min throughout and the injection volume was 8 μL. Under these conditions, **1** eluted at 13.8 min. The fraction containing **1** was concentrated at low pressure and lyophilized at high vacuum. A total of 11 mg was isolated as a clear glass; [α] 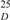: 0.0° (*c* 0.003 g/mL, MeOH); UV (MeOH) nm (log *ε*) = 311 (4.66); ^13^C- and ^1^H-NMR, see Table 1 HRFTICR MS *m/z* 707.40728 [M + H]^+^ calcd. for C_38_H_58_O_12_ (3.9 ppm error).

## 4. Conclusions

Diverging lineages of *P. subserratum* ecotypes found in the Amazon basin offer an intriguing glimpse into the process of habitat specialization We have found significant differences in the chemical composition of white-sand and clay ecotypes [8], including this newly described squalene derivative. We hypothesize that insect herbivory pressure is likely one of the mechanisms driving this divergence, and therefore the secondary metabolism of the plants that are preyed upon provides important clues as to how plants respond in evolutionary time to changes in herbivore communities. The occurrence of a modified form of an important primary metabolite, squalene, that we observe in higher concentrations in one ecotype of *P. subserratum*, is consistent with the hypothesis that insect herbivory can be a habitat-specific selective force. Future studies should investigate the effect of this squalene derivative on deterring specialist herbivores that occur on both white-sand and clay habitats.
